# Mg_20.66_Al_12.24_Zn_20.04_

**DOI:** 10.1107/S2414314625003062

**Published:** 2025-04-11

**Authors:** Jingchao Yu, Yibo Liu, Huizi Liu, Bin Wen, Lifeng Zhang, Changzeng Fan

**Affiliations:** a State Key Laboratory of Metastable Materials Science and Technology Yanshan University,Qinhuangdao 066004 People’s Republic of China; b School of Mechanical and Materials Engineering North China University of Technology,Beijing People’s Republic of China; c Hebei Key Lab for Optimizing Metal Product Technology and Performance Yanshan University,Qinhuangdao 066004 People’s Republic of China; University of Aberdeen, United Kingdom

**Keywords:** crystal structure, high-pressure sinter­ing, inter­metallic, Mg–Al–Zn phase

## Abstract

The title single-crystal (icosa­magnesium dodeca­aluminium icosa­zinc), was obtained during the synthesis of an Mg–Al–Zn alloy at high pressure and temperature. It crystallizes in space group *Im*3 (No. 204) with seven distinct metal-atom sites: three are occupied by aluminium and zinc, one by zinc and magnesium and three by magnesium (two partially occupied). One of the Al/Zn sites has icosa­hedral coordination. There are significant difference between the current model and that of previous studies [Montagné & Tillard (2016 ▸). *J. Alloys Compd.***656**, 159–165].

## Abstract 2

The title single-crystal (icosa­magnesium dodeca­aluminium icosa­zinc), was obtained during the synthesis of an Mg–Al–Zn alloy at high pressure and temperature. There are significant difference between the current model and that of previous studies.

## Graphical abstract

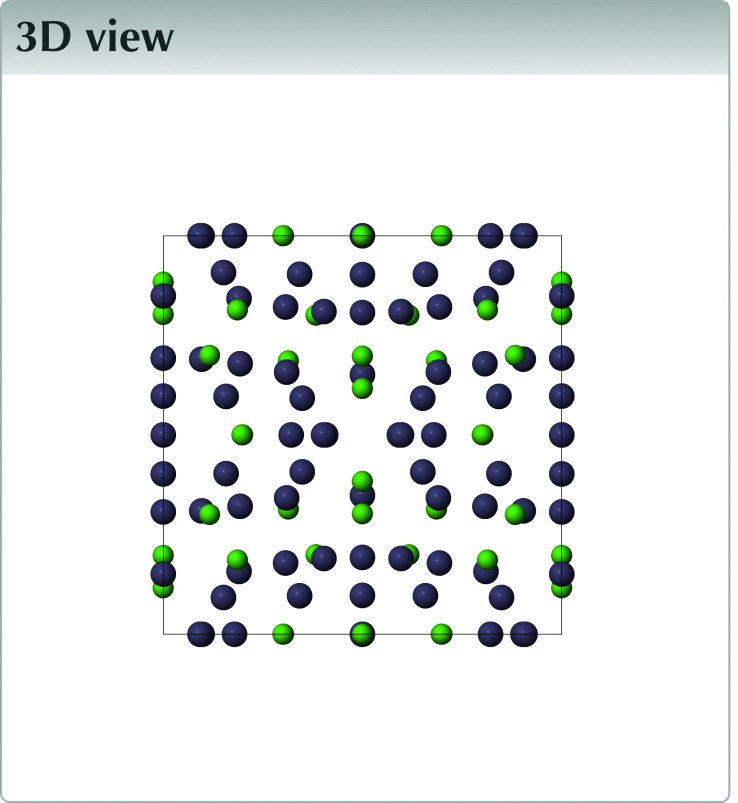

## Structure description

The discovery of quasicrystalline compounds in the Mg–Al–Zn systems has stimulated extensive studies (Berthold *et al.*, 2013 ▸). The quasicrystalline approximant phase with the composition Mg_32_Al_12_Zn_37_ is characterized by a low Al content (Montagné & Tillard, 2016 ▸). In a wider context, Mg–Al–Zn coatings are important materials for the corrosion protection of steel sheets. By the addition of some specific alloying elements including Al and Mg to zinc, the corrosion resistance and wear response of the zinc-based coatings are considerably enhanced (Yao *et al.*, 2016 ▸). The ratio of zinc to aluminium content is also of significance. Zn and Al are commonly used as alloying elements to enhance the mechanical properties of Mg alloys due to their strong solid solution-strengthening effects (Zhang *et al.*, 2022 ▸). As a result of proper Zn/Al ratio control, significant reduction, or elimination of the thermally unstable Mg_17_Al_12_ from the phase composition, Mg–Al–Zn (ZA) alloys are designed to demonstrate creep resistance and high-temperature mechanical capabilities (Edoziuno *et al.*, 2024 ▸).

In the present study, a cubic phase with *a* = 14.2100 (8) Å in space group *Im*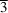 with composition Mg_20.66_Al_12.24_Zn_20.04_ has been established based on the refinement process by single-crystal X-ray diffraction, and its chemical composition is in accordance with the EDX results (see the supporting information).

**Figure 1:**
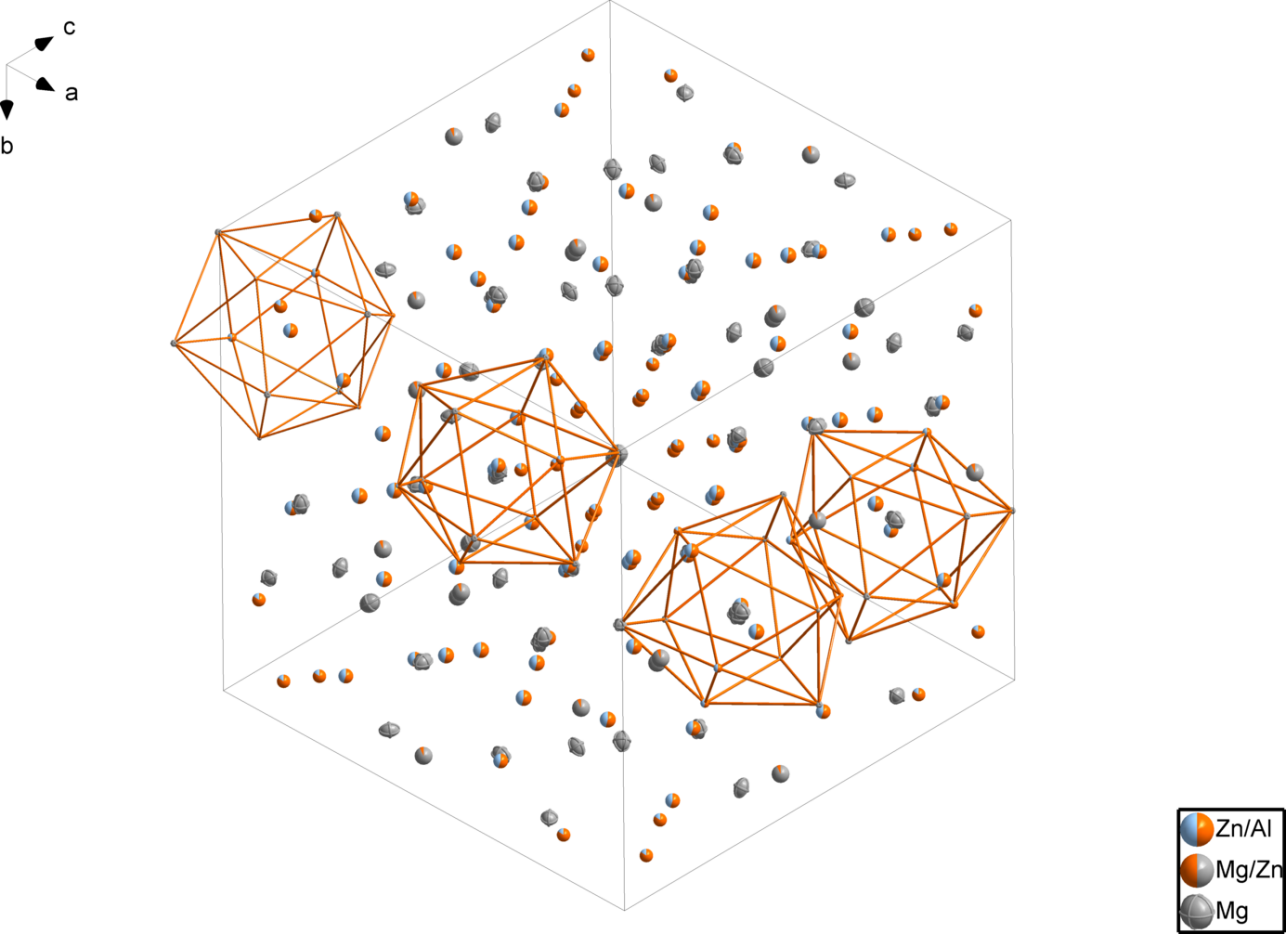
The crystal structure of Mg_20.66_Al_12.24_Zn_20.04_ (one unit cell), with displacement ellipsoids drawn at the 99% probability level.

**Figure 2:**
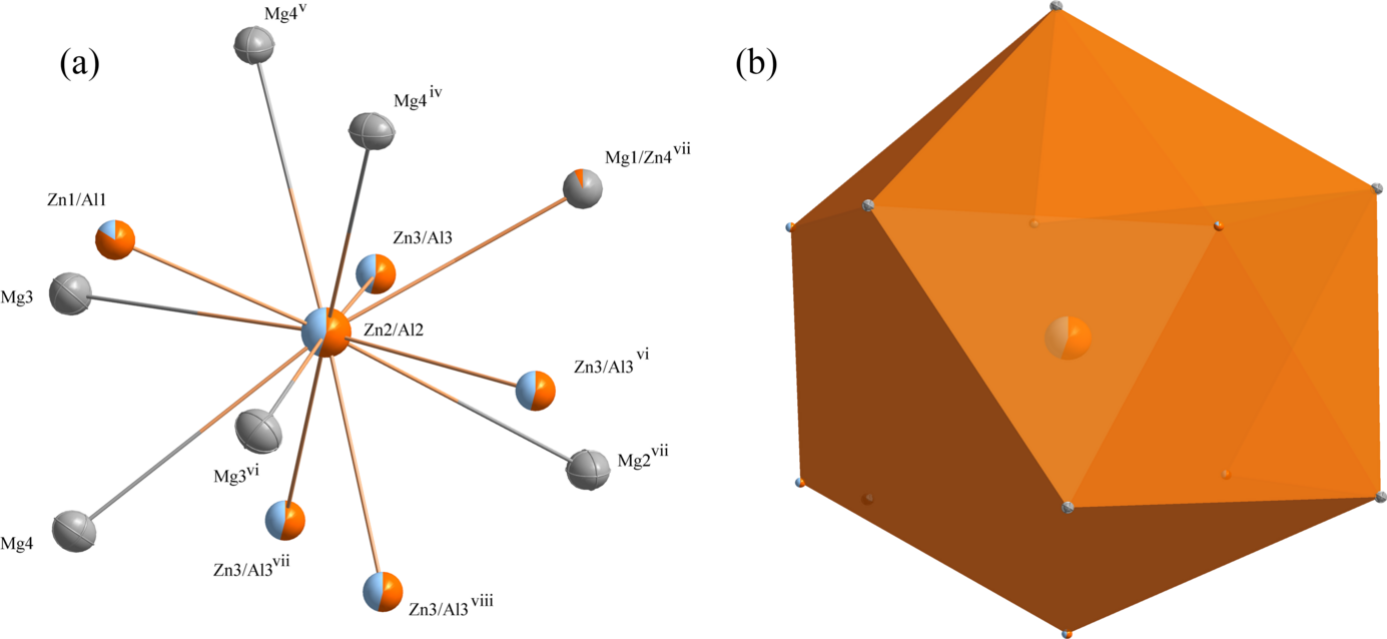
(*a*) the environment of the Zn2/Al2 atom with displacement ellipsoids given at the 99% probability level; (*b*) The icosa­hedron formed around the Zn2/Al2 atom at the 24 *g* site. [Symmetry codes: (iv) −*y*, *z*, −*x*; (v) *y*, *z*, *x*; (vi) −*x*, *y*, *z*; (vii) −*y* + 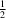, −*z* + 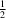, −*x* + 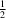; (viii) *y* − 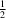, −*z* + 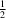, −*x* + 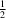.]

The unit cell is illustrated in Fig. 1 ▸. There are seven metal-atom sites: three are occupied by aluminium and zinc, one by zinc and magnesium and three by magnesium (two partially occupied). The environments of the Zn2/Al2 sites are delineated in Fig. 2 ▸. The Zn2/Al2 is located at a position with site symmetry *m*.. (multiplicity 24, Wyckoff letter *g*). The central Zn2/Al2 atom is surrounded by four Zn3/Al3 atoms (1, 48 *h*), two Mg3 atoms (.3., 16 *f*), one Mg2 atom (*mm*2.., 12 *e*), one Zn1/Al1 atom (*m*.., 24 *g*), one Mg1/Zn4 atom (*m*2.., 12 *e*), and three Mg4 atoms (*m*.., 24 *g*), which collectively define a icosa­hedron.

## Synthesis and crystallization

Magnesium (99.5% purity; 0.2241 g), aluminium (99.5% purity; 0.1125 g) and zinc (99.5% purity; 0.06633 g) were mixed in a stoichiometric ratio of 6:5:1 and ground in an agate mortar. Subsequently, the blended powder was placed in a carbide grinding die with a diameter of 5 mm and pressed into a tablet at approximately 4 MPa for 1 min to give a cylindrical block that exhibited no signs of deformation or cracking. Further details regarding the high-pressure sinter­ing experiment utilizing the hexa­nol high-temperature and high-pressure apparatus can be found in the published literature (Liu & Fan, 2018 ▸). The sample was subjected to a pressure of 4 GPa and heated to a temperature of 1073 K for a period of 30 min. The temperature was then reduced to 873 K and maintained for a further 30 min, before being rapidly cooled to room tem­perature by the deactivation of the furnace power. A grey single crystal was selected and mounted on glass fibres for SXRD measurement.

## Refinement

The crystal data, data collection and structure refinement details are outlined in Table 1 ▸. The Zn1 and Al1 atoms occupy a position in which the Zn1 atom occupancy is 0.840 (8) and the Al1 atom occupancy is 0.160 (8). Zn2 and Al2 atoms occupy a position in which the Zn2 atom occupancy is 0.550 (10) and the Al2 atom occupancy is 0.450 (10). The Zn3 and Al3 atoms coexist in a position where the Zn3 atom occupies 0.540 (8) and the Al3 atom occupies 0.460 (8). The Mg1 and Zn4 atoms coexist in a position where the Mg1 atom occupies 0.931 (3) and the Zn4 atom occupies 0.069 (3). The occupancy of the Mg2 atom is determined to be partial, with an occupancy number of 0.970 (3) whereas Mg3 is fully occupied. Finally, The Mg4 atom is also partially occupied, with an occupation number of 0.970 (2).

## Supplementary Material

Crystal structure: contains datablock(s) I. DOI: 10.1107/S2414314625003062/hb4508sup1.cif

Structure factors: contains datablock(s) I. DOI: 10.1107/S2414314625003062/hb4508Isup2.hkl

Supporting information file. DOI: 10.1107/S2414314625003062/hb4508sup3.docx

CCDC reference: 2440948

Additional supporting information:  crystallographic information; 3D view; checkCIF report

## Figures and Tables

**Table 1:** Experimental details

Crystal data	
Chemical formula	Mg_20.66_Al_12.24_Zn_20.04_
*M*_r_	2143.54
Crystal system, space group	Cubic, *Im* 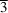
Temperature (K)	296
*a* (Å)	14.2100 (8)
*V* (Å^3^)	2869.3 (5)
*Z*	3
Radiation type	Mo *K*α
μ (mm^−1^)	12.91
Crystal size (mm)	0.08 × 0.06 × 0.06

Data collection	
Diffractometer	Bruker D8 Venture Photon 100 CMOS
Absorption correction	Multi-scan (*SADABS*; Krause *et al.*, 2015 ▸)
*T*_min_, *T*_max_	0.496, 0.523
No. of measured, independent and observed [*I* > 2σ(*I*)] reflections	6786, 481, 309
*R*_int_	0.180
(sin θ/λ)_max_ (Å^−1^)	0.595

Refinement	
*R*[*F*^2^ > 2σ(*F*^2^)], *wR*(*F*^2^), *S*	0.061, 0.129, 1.08
No. of reflections	481
No. of parameters	46
Δρ_max_, Δρ_min_ (e Å^−3^)	1.21, −0.89

Computer programs: *APEX3* and *SAINT* (Bruker, 2015 ▸), *SHELXT2018/2* (Sheldrick, 2015*a* ▸), *SHELXL2018/3* (Sheldrick, 2015*b* ▸), *DIAMOND* (Brandenburg & Putz, 2017 ▸) and *publCIF* (Westrip, 2010 ▸).

## full crystallographic data

### Icosamagnesium dodecaaluminium icosazinc Crystal data

**Table:**

Mg_20.66_Al_12.24_Zn_20.04_	Mo *K*α radiation, λ = 0.71073 Å
*M**_r_* = 2143.54	Cell parameters from 2565 reflections
Cubic, *I**m*3	θ = 3.2–27.0°
*a* = 14.2100 (8) Å	µ = 12.91 mm^−^^1^
*V* = 2869.3 (5) Å^3^	*T* = 296 K
*Z* = 3	Lump, gray
*F*(000) = 3024	0.08 × 0.06 × 0.06 mm
*D*_x_ = 3.719 Mg m^−^^3^	

### Icosamagnesium dodecaaluminium icosazinc Data collection

**Table:**

Bruker D8 Venture Photon 100 CMOS diffractometer	309 reflections with *I* > 2σ(*I*)
ω scans	*R*_int_ = 0.180
Absorption correction: multi-scan (SADABS; Krause *et al.*, 2015)	θ_max_ = 25.0°, θ_min_ = 2.9°
*T*_min_ = 0.496, *T*_max_ = 0.523	*h* = −15→16
6786 measured reflections	*k* = −12→16
481 independent reflections	*l* = −16→11

### Icosamagnesium dodecaaluminium icosazinc Refinement

**Table:**

Refinement on *F*^2^	Primary atom site location: dual
Least-squares matrix: full	*w* = 1/[σ^2^(*F*_o_^2^) + (0.0585*P*)^2^] where *P* = (*F*_o_^2^ + 2*F*_c_^2^)/3
*R*[*F*^2^ > 2σ(*F*^2^)] = 0.061	(Δ/σ)_max_ < 0.001
*wR*(*F*^2^) = 0.129	Δρ_max_ = 1.21 e Å^−^^3^
*S* = 1.08	Δρ_min_ = −0.89 e Å^−^^3^
481 reflections	Extinction correction: *SHELXL2018/3* (Sheldrick, 2015b), Fc^*^=kFc[1+0.001xFc^2^λ^3^/sin(2θ)]^-1/4^
46 parameters	Extinction coefficient: 0.00051 (13)
0 restraints	

### Icosamagnesium dodecaaluminium icosazinc Special details

**Table:**

Geometry. All esds (except the esd in the dihedral angle between two l.s. planes) are estimated using the full covariance matrix. The cell esds are taken into account individually in the estimation of esds in distances, angles and torsion angles; correlations between esds in cell parameters are only used when they are defined by crystal symmetry. An approximate (isotropic) treatment of cell esds is used for estimating esds involving l.s. planes.

### Icosamagnesium dodecaaluminium icosazinc Fractional atomic coordinates and isotropic or equivalent isotropic displacement parameters (Å^2^)

**Table:**

	*x*	*y*	*z*	*U*_iso_*/*U*_eq_	Occ. (<1)
Zn1	0.000000	0.15105 (13)	0.09251 (13)	0.0126 (8)	0.84 (3)
Al1	0.000000	0.15105 (13)	0.09251 (13)	0.0126 (8)	0.16 (3)
Zn2	0.000000	0.30698 (17)	0.17872 (18)	0.0151 (10)	0.55 (3)
Al2	0.000000	0.30698 (17)	0.17872 (18)	0.0151 (10)	0.45 (3)
Zn3	0.15796 (13)	0.40335 (12)	0.19039 (12)	0.0176 (8)	0.54 (3)
Al3	0.15796 (13)	0.40335 (12)	0.19039 (12)	0.0176 (8)	0.46 (3)
Mg1	0.4025 (5)	0.500000	0.000000	0.022 (3)	0.931 (17)
Zn4	0.4025 (5)	0.500000	0.000000	0.022 (3)	0.069 (17)
Mg2	0.1978 (5)	0.500000	0.000000	0.021 (3)	0.97 (3)
Mg3	0.1861 (3)	0.1861 (3)	0.1861 (3)	0.021 (3)	
Mg4	0.000000	0.1164 (4)	0.3006 (4)	0.018 (2)	0.97 (2)

### Icosamagnesium dodecaaluminium icosazinc Atomic displacement parameters (Å^2^)

**Table:**

	*U*^11^	*U*^22^	*U*^33^	*U*^12^	*U*^13^	*U*^23^
Zn1	0.0111 (12)	0.0147 (14)	0.0120 (13)	0.000	0.000	−0.0005 (9)
Al1	0.0111 (12)	0.0147 (14)	0.0120 (13)	0.000	0.000	−0.0005 (9)
Zn2	0.0154 (16)	0.0134 (16)	0.0166 (17)	0.000	0.000	−0.0035 (12)
Al2	0.0154 (16)	0.0134 (16)	0.0166 (17)	0.000	0.000	−0.0035 (12)
Zn3	0.0158 (13)	0.0166 (12)	0.0203 (12)	−0.0007 (9)	0.0011 (8)	0.0023 (8)
Al3	0.0158 (13)	0.0166 (12)	0.0203 (12)	−0.0007 (9)	0.0011 (8)	0.0023 (8)
Mg1	0.019 (5)	0.020 (5)	0.028 (5)	0.000	0.000	0.000
Zn4	0.019 (5)	0.020 (5)	0.028 (5)	0.000	0.000	0.000
Mg2	0.016 (5)	0.027 (6)	0.019 (6)	0.000	0.000	0.000
Mg3	0.021 (3)	0.021 (3)	0.021 (3)	0.0024 (18)	0.0024 (18)	0.0024 (18)
Mg4	0.014 (4)	0.021 (4)	0.019 (4)	0.000	0.000	0.004 (3)

### Icosamagnesium dodecaaluminium icosazinc Geometric parameters (Å, º)

**Table:**

Zn1—Zn2	2.532 (3)	Al2—Mg3^vi^	3.1553 (19)
Zn1—Zn1^i^	2.629 (4)	Al2—Mg4	3.214 (6)
Zn1—Zn1^ii^	2.651 (2)	Al2—Mg2^vii^	3.256 (5)
Zn1—Zn1^iii^	2.651 (2)	Zn3—Zn3^vii^	2.6790 (18)
Zn1—Zn1^iv^	2.651 (2)	Zn3—Zn3^ix^	2.6791 (18)
Zn1—Zn1^v^	2.651 (2)	Zn3—Zn3^x^	2.747 (3)
Zn1—Mg4^v^	2.997 (5)	Zn3—Mg1^vii^	2.944 (3)
Zn1—Mg4^iv^	2.997 (5)	Zn3—Mg2^vii^	3.074 (4)
Zn1—Mg4	2.998 (6)	Zn3—Mg2	3.087 (2)
Zn1—Mg3	3.002 (6)	Zn3—Mg3^xi^	3.099 (4)
Zn1—Mg3^vi^	3.002 (6)	Zn3—Mg3	3.113 (4)
Al1—Al2	2.532 (3)	Zn3—Mg4^ix^	3.125 (5)
Al1—Mg4^v^	2.997 (5)	Zn3—Mg4^v^	3.130 (3)
Al1—Mg4^iv^	2.997 (5)	Al3—Mg2^vii^	3.074 (4)
Al1—Mg4	2.998 (6)	Al3—Mg2	3.087 (2)
Al1—Mg3	3.002 (6)	Al3—Mg3^xi^	3.099 (4)
Al1—Mg3^vi^	3.002 (6)	Al3—Mg3	3.113 (4)
Zn2—Zn3	2.635 (2)	Al3—Mg4^ix^	3.125 (5)
Zn2—Zn3^vi^	2.635 (2)	Al3—Mg4^v^	3.130 (3)
Zn2—Zn3^vii^	2.697 (3)	Mg1—Mg1^xii^	2.771 (13)
Zn2—Zn3^viii^	2.697 (3)	Mg1—Mg2	2.909 (10)
Zn2—Mg1^vii^	2.976 (4)	Mg1—Mg2^xiii^	3.134 (7)
Zn2—Mg4^iv^	3.032 (4)	Mg1—Mg2^ix^	3.134 (7)
Zn2—Mg4^v^	3.032 (4)	Mg1—Mg4^xiv^	3.562 (5)
Zn2—Mg3	3.1553 (19)	Mg1—Mg4^xv^	3.562 (5)
Zn2—Mg3^vi^	3.1553 (19)	Zn4—Mg2	2.909 (10)
Zn2—Mg4	3.214 (6)	Zn4—Mg2^xiii^	3.134 (7)
Zn2—Mg2^vii^	3.256 (5)	Zn4—Mg2^ix^	3.134 (7)
Al2—Al3	2.635 (2)	Mg2—Mg4^v^	3.060 (6)
Al2—Mg4^iv^	3.032 (4)	Mg2—Mg4^xvi^	3.060 (6)
Al2—Mg4^v^	3.032 (4)	Mg3—Mg3^xi^	3.144 (13)
Al2—Mg3	3.1553 (19)		
			
Zn2—Zn1—Zn1^i^	118.94 (7)	Mg1^xii^—Mg1—Mg2	180.0
Zn2—Zn1—Zn1^ii^	123.83 (7)	Mg1^xii^—Mg1—Zn3^xvii^	116.64 (12)
Zn1^i^—Zn1—Zn1^ii^	108.29 (5)	Mg2—Mg1—Zn3^xvii^	63.36 (12)
Zn2—Zn1—Zn1^iii^	123.83 (7)	Mg1^xii^—Mg1—Zn3^xviii^	116.64 (12)
Zn1^i^—Zn1—Zn1^iii^	108.29 (5)	Mg2—Mg1—Zn3^xviii^	63.36 (12)
Zn1^ii^—Zn1—Zn1^iii^	59.45 (10)	Zn3^xvii^—Mg1—Zn3^xviii^	126.7 (2)
Zn2—Zn1—Zn1^iv^	120.97 (4)	Mg1^xii^—Mg1—Zn3^ix^	116.64 (12)
Zn1^i^—Zn1—Zn1^iv^	60.27 (5)	Mg2—Mg1—Zn3^ix^	63.36 (12)
Zn1^ii^—Zn1—Zn1^iv^	60.0	Zn3^xvii^—Mg1—Zn3^ix^	55.62 (9)
Zn1^iii^—Zn1—Zn1^iv^	107.64 (6)	Zn3^xviii^—Mg1—Zn3^ix^	99.36 (15)
Zn2—Zn1—Zn1^v^	120.97 (4)	Mg1^xii^—Mg1—Zn3^xix^	116.64 (12)
Zn1^i^—Zn1—Zn1^v^	60.27 (5)	Mg2—Mg1—Zn3^xix^	63.36 (12)
Zn1^ii^—Zn1—Zn1^v^	107.64 (6)	Zn3^xvii^—Mg1—Zn3^xix^	99.36 (15)
Zn1^iii^—Zn1—Zn1^v^	60.0	Zn3^xviii^—Mg1—Zn3^xix^	55.62 (9)
Zn1^iv^—Zn1—Zn1^v^	108.13 (2)	Zn3^ix^—Mg1—Zn3^xix^	126.7 (2)
Zn2—Zn1—Mg4^v^	65.90 (10)	Mg1^xii^—Mg1—Zn2^xix^	112.82 (13)
Zn1^i^—Zn1—Mg4^v^	63.98 (6)	Mg2—Mg1—Zn2^xix^	67.18 (13)
Zn1^ii^—Zn1—Mg4^v^	170.25 (9)	Zn3^xvii^—Mg1—Zn2^xix^	52.85 (6)
Zn1^iii^—Zn1—Mg4^v^	115.98 (11)	Zn3^xviii^—Mg1—Zn2^xix^	104.84 (14)
Zn1^iv^—Zn1—Mg4^v^	116.87 (11)	Zn3^ix^—Mg1—Zn2^xix^	104.84 (14)
Zn1^v^—Zn1—Mg4^v^	63.77 (12)	Zn3^xix^—Mg1—Zn2^xix^	52.85 (6)
Zn2—Zn1—Mg4^iv^	65.90 (10)	Mg1^xii^—Mg1—Zn2^ix^	112.82 (13)
Zn1^i^—Zn1—Mg4^iv^	63.98 (6)	Mg2—Mg1—Zn2^ix^	67.18 (13)
Zn1^ii^—Zn1—Mg4^iv^	115.98 (11)	Zn3^xvii^—Mg1—Zn2^ix^	104.84 (14)
Zn1^iii^—Zn1—Mg4^iv^	170.25 (9)	Zn3^xviii^—Mg1—Zn2^ix^	52.85 (6)
Zn1^iv^—Zn1—Mg4^iv^	63.77 (12)	Zn3^ix^—Mg1—Zn2^ix^	52.85 (6)
Zn1^v^—Zn1—Mg4^iv^	116.87 (11)	Zn3^xix^—Mg1—Zn2^ix^	104.84 (14)
Mg4^v^—Zn1—Mg4^iv^	67.0 (2)	Zn2^xix^—Mg1—Zn2^ix^	134.4 (3)
Zn2—Zn1—Mg4	70.51 (12)	Mg1^xii^—Mg1—Mg2^xiii^	63.76 (12)
Zn1^i^—Zn1—Mg4	170.56 (11)	Mg2—Mg1—Mg2^xiii^	116.24 (12)
Zn1^ii^—Zn1—Mg4	63.74 (12)	Zn3^xvii^—Mg1—Mg2^xiii^	151.90 (3)
Zn1^iii^—Zn1—Mg4	63.74 (11)	Zn3^xviii^—Mg1—Mg2^xiii^	60.94 (6)
Zn1^iv^—Zn1—Mg4	115.95 (8)	Zn3^ix^—Mg1—Mg2^xiii^	151.90 (3)
Zn1^v^—Zn1—Mg4	115.95 (8)	Zn3^xix^—Mg1—Mg2^xiii^	60.94 (6)
Mg4^v^—Zn1—Mg4	123.30 (13)	Zn2^xix^—Mg1—Mg2^xiii^	99.87 (3)
Mg4^iv^—Zn1—Mg4	123.30 (13)	Zn2^ix^—Mg1—Mg2^xiii^	99.87 (3)
Zn2—Zn1—Mg3	68.92 (7)	Mg1^xii^—Mg1—Mg2^ix^	63.76 (12)
Zn1^i^—Zn1—Mg3	116.30 (4)	Mg2—Mg1—Mg2^ix^	116.24 (12)
Zn1^ii^—Zn1—Mg3	115.61 (10)	Zn3^xvii^—Mg1—Mg2^ix^	60.94 (6)
Zn1^iii^—Zn1—Mg3	63.80 (6)	Zn3^xviii^—Mg1—Mg2^ix^	151.90 (3)
Zn1^iv^—Zn1—Mg3	170.11 (8)	Zn3^ix^—Mg1—Mg2^ix^	60.94 (6)
Zn1^v^—Zn1—Mg3	63.80 (6)	Zn3^xix^—Mg1—Mg2^ix^	151.90 (3)
Mg4^v^—Zn1—Mg3	65.81 (8)	Zn2^xix^—Mg1—Mg2^ix^	99.87 (3)
Mg4^iv^—Zn1—Mg3	124.26 (13)	Zn2^ix^—Mg1—Mg2^ix^	99.87 (3)
Mg4—Zn1—Mg3	65.80 (4)	Mg2^xiii^—Mg1—Mg2^ix^	127.5 (2)
Zn2—Zn1—Mg3^vi^	68.92 (7)	Mg1^xii^—Mg1—Mg4^xiv^	67.11 (11)
Zn1^i^—Zn1—Mg3^vi^	116.30 (4)	Mg2—Mg1—Mg4^xiv^	112.89 (11)
Zn1^ii^—Zn1—Mg3^vi^	63.80 (6)	Zn3^xvii^—Mg1—Mg4^xiv^	154.12 (10)
Zn1^iii^—Zn1—Mg3^vi^	115.61 (10)	Zn3^xviii^—Mg1—Mg4^xiv^	56.57 (6)
Zn1^iv^—Zn1—Mg3^vi^	63.80 (6)	Zn3^ix^—Mg1—Mg4^xiv^	99.06 (9)
Zn1^v^—Zn1—Mg3^vi^	170.11 (8)	Zn3^xix^—Mg1—Mg4^xiv^	101.03 (9)
Mg4^v^—Zn1—Mg3^vi^	124.26 (13)	Zn2^xix^—Mg1—Mg4^xiv^	152.15 (9)
Mg4^iv^—Zn1—Mg3^vi^	65.81 (8)	Zn2^ix^—Mg1—Mg4^xiv^	54.38 (7)
Mg4—Zn1—Mg3^vi^	65.80 (4)	Mg2^xiii^—Mg1—Mg4^xiv^	53.94 (10)
Mg3—Zn1—Mg3^vi^	123.52 (12)	Mg2^ix^—Mg1—Mg4^xiv^	104.16 (15)
Al2—Al1—Mg4^v^	65.90 (10)	Mg1^xii^—Mg1—Mg4^xv^	67.11 (11)
Al2—Al1—Mg4^iv^	65.90 (10)	Mg2—Mg1—Mg4^xv^	112.89 (11)
Mg4^v^—Al1—Mg4^iv^	67.0 (2)	Zn3^xvii^—Mg1—Mg4^xv^	56.57 (6)
Al2—Al1—Mg4	70.51 (12)	Zn3^xviii^—Mg1—Mg4^xv^	154.12 (10)
Mg4^v^—Al1—Mg4	123.30 (13)	Zn3^ix^—Mg1—Mg4^xv^	101.03 (9)
Mg4^iv^—Al1—Mg4	123.30 (13)	Zn3^xix^—Mg1—Mg4^xv^	99.06 (9)
Al2—Al1—Mg3	68.92 (7)	Zn2^xix^—Mg1—Mg4^xv^	54.38 (7)
Mg4^v^—Al1—Mg3	65.81 (8)	Zn2^ix^—Mg1—Mg4^xv^	152.15 (9)
Mg4^iv^—Al1—Mg3	124.26 (13)	Mg2^xiii^—Mg1—Mg4^xv^	104.16 (15)
Mg4—Al1—Mg3	65.80 (4)	Mg2^ix^—Mg1—Mg4^xv^	53.94 (10)
Mg4^v^—Al1—Mg3^vi^	124.26 (13)	Mg4^xiv^—Mg1—Mg4^xv^	134.2 (2)
Mg4^iv^—Al1—Mg3^vi^	65.81 (8)	Mg2—Zn4—Mg2^xiii^	116.24 (12)
Mg4—Al1—Mg3^vi^	65.80 (4)	Mg2—Zn4—Mg2^ix^	116.24 (12)
Mg3—Al1—Mg3^vi^	123.52 (12)	Mg2^xiii^—Zn4—Mg2^ix^	127.5 (2)
Zn1—Zn2—Zn3	119.04 (7)	Zn4—Mg2—Mg4^v^	112.20 (17)
Zn1—Zn2—Zn3^vi^	119.04 (7)	Mg1—Mg2—Mg4^v^	112.20 (17)
Zn3—Zn2—Zn3^vi^	116.85 (12)	Zn4—Mg2—Mg4^xvi^	112.20 (17)
Zn1—Zn2—Zn3^vii^	115.37 (9)	Mg1—Mg2—Mg4^xvi^	112.20 (17)
Zn3—Zn2—Zn3^vii^	60.31 (5)	Mg4^v^—Mg2—Mg4^xvi^	135.6 (3)
Zn3^vi^—Zn2—Zn3^vii^	111.87 (10)	Zn4—Mg2—Zn3^xviii^	58.88 (12)
Zn1—Zn2—Zn3^viii^	115.37 (9)	Mg1—Mg2—Zn3^xviii^	58.88 (12)
Zn3—Zn2—Zn3^viii^	111.87 (10)	Mg4^v^—Mg2—Zn3^xviii^	150.62 (9)
Zn3^vi^—Zn2—Zn3^viii^	60.32 (5)	Mg4^xvi^—Mg2—Zn3^xviii^	61.26 (10)
Zn3^vii^—Zn2—Zn3^viii^	61.22 (10)	Zn4—Mg2—Zn3^xix^	58.88 (12)
Zn1—Zn2—Mg1^vii^	128.24 (15)	Mg1—Mg2—Zn3^xix^	58.88 (12)
Zn3—Zn2—Mg1^vii^	62.96 (7)	Mg4^v^—Mg2—Zn3^xix^	150.62 (9)
Zn3^vi^—Zn2—Mg1^vii^	62.96 (7)	Mg4^xvi^—Mg2—Zn3^xix^	61.26 (10)
Zn3^vii^—Zn2—Mg1^vii^	108.72 (12)	Zn3^xviii^—Mg2—Zn3^xix^	53.08 (9)
Zn3^viii^—Zn2—Mg1^vii^	108.72 (12)	Zn4—Mg2—Zn3^xvii^	58.88 (12)
Zn1—Zn2—Mg4^iv^	64.44 (12)	Mg1—Mg2—Zn3^xvii^	58.88 (12)
Zn3—Zn2—Mg4^iv^	122.22 (13)	Mg4^v^—Mg2—Zn3^xvii^	61.26 (10)
Zn3^vi^—Zn2—Mg4^iv^	66.64 (10)	Mg4^xvi^—Mg2—Zn3^xvii^	150.62 (9)
Zn3^vii^—Zn2—Mg4^iv^	177.37 (12)	Zn3^xviii^—Mg2—Zn3^xvii^	117.8 (2)
Zn3^viii^—Zn2—Mg4^iv^	116.31 (9)	Zn3^xix^—Mg2—Zn3^xvii^	93.81 (17)
Mg1^vii^—Zn2—Mg4^iv^	72.71 (14)	Zn4—Mg2—Zn3^ix^	58.88 (12)
Zn1—Zn2—Mg4^v^	64.44 (12)	Mg1—Mg2—Zn3^ix^	58.88 (12)
Zn3—Zn2—Mg4^v^	66.64 (10)	Mg4^v^—Mg2—Zn3^ix^	61.26 (10)
Zn3^vi^—Zn2—Mg4^v^	122.22 (13)	Mg4^xvi^—Mg2—Zn3^ix^	150.62 (9)
Zn3^vii^—Zn2—Mg4^v^	116.31 (9)	Zn3^xviii^—Mg2—Zn3^ix^	93.81 (17)
Zn3^viii^—Zn2—Mg4^v^	177.37 (12)	Zn3^xix^—Mg2—Zn3^ix^	117.8 (2)
Mg1^vii^—Zn2—Mg4^v^	72.71 (14)	Zn3^xvii^—Mg2—Zn3^ix^	53.08 (9)
Mg4^iv^—Zn2—Mg4^v^	66.13 (18)	Mg1—Mg2—Zn3^i^	100.57 (14)
Zn1—Zn2—Mg3	62.60 (13)	Mg4^v^—Mg2—Zn3^i^	61.23 (6)
Zn3—Zn2—Mg3	64.32 (11)	Mg4^xvi^—Mg2—Zn3^i^	110.04 (12)
Zn3^vi^—Zn2—Mg3	174.25 (12)	Zn3^xviii^—Mg2—Zn3^i^	144.24 (15)
Zn3^vii^—Zn2—Mg3	63.38 (11)	Zn3^xix^—Mg2—Zn3^i^	91.61 (5)
Zn3^viii^—Zn2—Mg3	113.93 (10)	Zn3^xvii^—Mg2—Zn3^i^	51.56 (6)
Mg1^vii^—Zn2—Mg3	120.97 (7)	Zn3^ix^—Mg2—Zn3^i^	99.30 (6)
Mg4^iv^—Zn2—Mg3	117.97 (16)	Mg1—Mg2—Zn3^x^	100.57 (14)
Mg4^v^—Zn2—Mg3	63.53 (10)	Mg4^v^—Mg2—Zn3^x^	110.04 (12)
Zn1—Zn2—Mg3^vi^	62.60 (13)	Mg4^xvi^—Mg2—Zn3^x^	61.23 (6)
Zn3—Zn2—Mg3^vi^	174.25 (12)	Zn3^xviii^—Mg2—Zn3^x^	51.56 (6)
Zn3^vi^—Zn2—Mg3^vi^	64.32 (11)	Zn3^xix^—Mg2—Zn3^x^	99.30 (6)
Zn3^vii^—Zn2—Mg3^vi^	113.93 (10)	Zn3^xvii^—Mg2—Zn3^x^	144.24 (15)
Zn3^viii^—Zn2—Mg3^vi^	63.38 (11)	Zn3^ix^—Mg2—Zn3^x^	91.61 (5)
Mg1^vii^—Zn2—Mg3^vi^	120.97 (7)	Zn3^i^—Mg2—Zn3^x^	158.9 (3)
Mg4^iv^—Zn2—Mg3^vi^	63.53 (10)	Mg1—Mg2—Zn3^xx^	100.57 (14)
Mg4^v^—Zn2—Mg3^vi^	117.97 (16)	Mg4^v^—Mg2—Zn3^xx^	110.04 (12)
Mg3—Zn2—Mg3^vi^	113.9 (2)	Mg4^xvi^—Mg2—Zn3^xx^	61.23 (6)
Zn1—Zn2—Mg4	61.54 (11)	Zn3^xviii^—Mg2—Zn3^xx^	99.30 (6)
Zn3—Zn2—Mg4	113.83 (8)	Zn3^xix^—Mg2—Zn3^xx^	51.56 (6)
Zn3^vi^—Zn2—Mg4	113.82 (8)	Zn3^xvii^—Mg2—Zn3^xx^	91.61 (5)
Zn3^vii^—Zn2—Mg4	63.13 (10)	Zn3^ix^—Mg2—Zn3^xx^	144.24 (15)
Zn3^viii^—Zn2—Mg4	63.13 (10)	Zn3^i^—Mg2—Zn3^xx^	52.84 (7)
Mg1^vii^—Zn2—Mg4	170.22 (18)	Zn3^x^—Mg2—Zn3^xx^	122.46 (11)
Mg4^iv^—Zn2—Mg4	115.22 (17)	Mg1—Mg2—Zn3	100.57 (14)
Mg4^v^—Zn2—Mg4	115.22 (17)	Mg4^v^—Mg2—Zn3	61.23 (6)
Mg3—Zn2—Mg4	61.54 (6)	Mg4^xvi^—Mg2—Zn3	110.04 (12)
Mg3^vi^—Zn2—Mg4	61.55 (6)	Zn3^xviii^—Mg2—Zn3	91.61 (5)
Zn1—Zn2—Mg2^vii^	176.33 (15)	Zn3^xix^—Mg2—Zn3	144.23 (15)
Zn3—Zn2—Mg2^vii^	61.85 (7)	Zn3^xvii^—Mg2—Zn3	99.30 (6)
Zn3^vi^—Zn2—Mg2^vii^	61.85 (7)	Zn3^ix^—Mg2—Zn3	51.56 (6)
Zn3^vii^—Zn2—Mg2^vii^	61.62 (11)	Zn3^i^—Mg2—Zn3	122.46 (11)
Zn3^viii^—Zn2—Mg2^vii^	61.62 (11)	Zn3^x^—Mg2—Zn3	52.84 (7)
Mg1^vii^—Zn2—Mg2^vii^	55.43 (17)	Zn3^xx^—Mg2—Zn3	158.9 (3)
Mg4^iv^—Zn2—Mg2^vii^	118.46 (14)	Al1—Mg3—Zn1^v^	52.40 (12)
Mg4^v^—Zn2—Mg2^vii^	118.46 (14)	Zn1—Mg3—Zn1^v^	52.40 (12)
Mg3—Zn2—Mg2^vii^	116.13 (13)	Al1—Mg3—Zn1^iii^	52.40 (12)
Mg3^vi^—Zn2—Mg2^vii^	116.13 (13)	Zn1—Mg3—Zn1^iii^	52.40 (12)
Mg4—Zn2—Mg2^vii^	114.79 (17)	Zn1^v^—Mg3—Zn1^iii^	52.40 (12)
Al1—Al2—Al3	119.04 (7)	Al1—Mg3—Zn3^vii^	92.83 (7)
Al1—Al2—Mg4^iv^	64.44 (12)	Zn1—Mg3—Zn3^vii^	92.83 (7)
Al3—Al2—Mg4^iv^	122.22 (13)	Zn1^v^—Mg3—Zn3^vii^	144.35 (15)
Al1—Al2—Mg4^v^	64.44 (12)	Zn1^iii^—Mg3—Zn3^vii^	115.83 (9)
Al3—Al2—Mg4^v^	66.64 (10)	Al1—Mg3—Zn3^ix^	115.83 (9)
Mg4^iv^—Al2—Mg4^v^	66.13 (18)	Zn1—Mg3—Zn3^ix^	115.83 (9)
Al1—Al2—Mg3	62.60 (13)	Zn1^v^—Mg3—Zn3^ix^	92.83 (7)
Al3—Al2—Mg3	64.32 (11)	Zn1^iii^—Mg3—Zn3^ix^	144.35 (15)
Mg4^iv^—Al2—Mg3	117.97 (16)	Zn3^vii^—Mg3—Zn3^ix^	96.94 (15)
Mg4^v^—Al2—Mg3	63.53 (10)	Zn1—Mg3—Zn3^xi^	144.35 (15)
Mg4^iv^—Al2—Mg3^vi^	63.53 (10)	Zn1^v^—Mg3—Zn3^xi^	115.83 (9)
Mg4^v^—Al2—Mg3^vi^	117.97 (16)	Zn1^iii^—Mg3—Zn3^xi^	92.83 (7)
Mg3—Al2—Mg3^vi^	113.9 (2)	Zn3^vii^—Mg3—Zn3^xi^	96.94 (15)
Al1—Al2—Mg4	61.54 (11)	Zn3^ix^—Mg3—Zn3^xi^	96.94 (15)
Al3—Al2—Mg4	113.83 (8)	Al1—Mg3—Zn3^v^	145.06 (15)
Mg4^iv^—Al2—Mg4	115.22 (17)	Zn1—Mg3—Zn3^v^	145.06 (15)
Mg4^v^—Al2—Mg4	115.22 (17)	Zn1^v^—Mg3—Zn3^v^	93.44 (7)
Mg3—Al2—Mg4	61.54 (6)	Zn1^iii^—Mg3—Zn3^v^	115.79 (8)
Mg3^vi^—Al2—Mg4	61.55 (6)	Zn3^vii^—Mg3—Zn3^v^	119.2 (2)
Mg4^iv^—Al2—Mg2^vii^	118.46 (14)	Zn3^ix^—Mg3—Zn3^v^	51.09 (6)
Mg4^v^—Al2—Mg2^vii^	118.46 (14)	Zn3^xi^—Mg3—Zn3^v^	51.09 (6)
Mg3—Al2—Mg2^vii^	116.13 (13)	Al1—Mg3—Zn3^iii^	115.79 (8)
Mg3^vi^—Al2—Mg2^vii^	116.13 (13)	Zn1—Mg3—Zn3^iii^	115.79 (8)
Mg4—Al2—Mg2^vii^	114.79 (17)	Zn1^v^—Mg3—Zn3^iii^	145.06 (15)
Zn2—Zn3—Zn3^vii^	61.00 (9)	Zn1^iii^—Mg3—Zn3^iii^	93.44 (7)
Zn2—Zn3—Zn3^ix^	119.01 (11)	Zn3^vii^—Mg3—Zn3^iii^	51.09 (6)
Zn3^vii^—Zn3—Zn3^ix^	119.988 (3)	Zn3^ix^—Mg3—Zn3^iii^	119.2 (2)
Zn2—Zn3—Zn2^ix^	177.08 (10)	Zn3^xi^—Mg3—Zn3^iii^	51.09 (6)
Zn3^vii^—Zn3—Zn2^ix^	121.46 (10)	Zn3^v^—Mg3—Zn3^iii^	96.36 (15)
Zn3^ix^—Zn3—Zn2^ix^	58.69 (8)	Zn1—Mg3—Zn3	93.44 (7)
Zn2—Zn3—Zn3^x^	121.32 (6)	Zn1^v^—Mg3—Zn3	115.79 (8)
Zn3^vii^—Zn3—Zn3^x^	119.82 (6)	Zn1^iii^—Mg3—Zn3	145.06 (15)
Zn3^ix^—Zn3—Zn3^x^	108.98 (5)	Zn3^vii^—Mg3—Zn3	51.09 (6)
Zn2^ix^—Zn3—Zn3^x^	59.39 (5)	Zn3^ix^—Mg3—Zn3	51.10 (6)
Zn2—Zn3—Mg1^vii^	64.19 (7)	Zn3^xi^—Mg3—Zn3	119.2 (2)
Zn3^vii^—Zn3—Mg1^vii^	110.16 (12)	Zn3^v^—Mg3—Zn3	96.36 (15)
Zn3^ix^—Zn3—Mg1^vii^	122.82 (14)	Zn3^iii^—Mg3—Zn3	96.36 (15)
Zn2^ix^—Zn3—Mg1^vii^	115.14 (7)	Al1—Mg3—Al3	93.44 (7)
Zn3^x^—Zn3—Mg1^vii^	62.19 (4)	Zn1—Mg3—Mg3^xi^	149.35 (7)
Zn2—Zn3—Mg2^vii^	69.06 (8)	Zn1^v^—Mg3—Mg3^xi^	149.35 (7)
Zn3^vii^—Zn3—Mg2^vii^	64.47 (11)	Zn1^iii^—Mg3—Mg3^xi^	149.35 (7)
Zn3^ix^—Zn3—Mg2^vii^	171.76 (10)	Zn3^vii^—Mg3—Mg3^xi^	59.82 (11)
Zn2^ix^—Zn3—Mg2^vii^	113.19 (8)	Zn3^ix^—Mg3—Mg3^xi^	59.82 (11)
Zn3^x^—Zn3—Mg2^vii^	63.46 (5)	Zn3^xi^—Mg3—Mg3^xi^	59.82 (11)
Mg1^vii^—Zn3—Mg2^vii^	57.76 (17)	Zn3^v^—Mg3—Mg3^xi^	59.37 (11)
Zn2—Zn3—Mg2	109.42 (14)	Zn3^iii^—Mg3—Mg3^xi^	59.37 (11)
Zn3^vii^—Zn3—Mg2	170.40 (16)	Zn3—Mg3—Mg3^xi^	59.37 (11)
Zn3^ix^—Zn3—Mg2	63.98 (13)	Zn1^ii^—Mg4—Zn1^iii^	52.03 (11)
Zn2^ix^—Zn3—Mg2	68.14 (13)	Zn1^ii^—Mg4—Zn1	52.49 (11)
Zn3^x^—Zn3—Mg2	63.58 (4)	Zn1^iii^—Mg4—Zn1	52.49 (11)
Mg1^vii^—Zn3—Mg2	62.57 (17)	Zn1^ii^—Mg4—Zn2^ii^	49.65 (9)
Mg2^vii^—Zn3—Mg2	112.84 (5)	Zn1^iii^—Mg4—Zn2^ii^	95.01 (15)
Zn2—Zn3—Mg3^xi^	115.56 (11)	Zn1—Mg4—Zn2^ii^	96.84 (13)
Zn3^vii^—Zn3—Mg3^xi^	64.72 (5)	Al1—Mg4—Zn2^ii^	96.84 (13)
Zn3^ix^—Zn3—Mg3^xi^	64.72 (5)	Zn1^ii^—Mg4—Zn2^iii^	95.01 (15)
Zn2^ix^—Zn3—Mg3^xi^	65.54 (10)	Zn1^iii^—Mg4—Zn2^iii^	49.65 (9)
Zn3^x^—Zn3—Mg3^xi^	114.23 (11)	Zn1—Mg4—Zn2^iii^	96.84 (13)
Mg1^vii^—Zn3—Mg3^xi^	172.04 (15)	Al1—Mg4—Zn2^iii^	96.84 (13)
Mg2^vii^—Zn3—Mg3^xi^	114.38 (14)	Zn2^ii^—Mg4—Zn2^iii^	113.75 (18)
Mg2—Zn3—Mg3^xi^	123.21 (14)	Zn1^ii^—Mg4—Mg2^iii^	149.90 (13)
Zn2—Zn3—Mg3	65.98 (10)	Zn1^iii^—Mg4—Mg2^iii^	149.90 (13)
Zn3^vii^—Zn3—Mg3	64.18 (5)	Zn1—Mg4—Mg2^iii^	148.4 (3)
Zn3^ix^—Zn3—Mg3	64.18 (5)	Al1—Mg4—Mg2^iii^	148.4 (3)
Zn2^ix^—Zn3—Mg3	113.23 (11)	Zn2^ii^—Mg4—Mg2^iii^	100.28 (14)
Zn3^x^—Zn3—Mg3	172.53 (8)	Zn2^iii^—Mg4—Mg2^iii^	100.28 (14)
Mg1^vii^—Zn3—Mg3	123.50 (12)	Zn1^ii^—Mg4—Zn3^vii^	144.22 (19)
Mg2^vii^—Zn3—Mg3	123.16 (7)	Zn1^iii^—Mg4—Zn3^vii^	115.20 (10)
Mg2—Zn3—Mg3	113.61 (8)	Zn1—Mg4—Zn3^vii^	92.39 (15)
Mg3^xi^—Zn3—Mg3	60.8 (2)	Al1—Mg4—Zn3^vii^	92.39 (15)
Zn2—Zn3—Mg4^ix^	116.38 (12)	Zn2^ii^—Mg4—Zn3^vii^	147.31 (15)
Zn3^vii^—Zn3—Mg4^ix^	64.74 (10)	Zn2^iii^—Mg4—Zn3^vii^	96.06 (6)
Zn3^ix^—Zn3—Mg4^ix^	115.34 (13)	Mg2^iii^—Mg4—Zn3^vii^	59.58 (16)
Zn2^ix^—Zn3—Mg4^ix^	66.54 (11)	Zn1^ii^—Mg4—Zn3^viii^	115.20 (10)
Zn3^x^—Zn3—Mg4^ix^	63.93 (5)	Zn1^iii^—Mg4—Zn3^viii^	144.22 (19)
Mg1^vii^—Zn3—Mg4^ix^	109.42 (14)	Zn1—Mg4—Zn3^viii^	92.39 (15)
Mg2^vii^—Zn3—Mg4^ix^	59.16 (14)	Zn2^ii^—Mg4—Zn3^viii^	96.06 (6)
Mg2—Zn3—Mg4^ix^	122.67 (9)	Zn2^iii^—Mg4—Zn3^viii^	147.31 (15)
Mg3^xi^—Zn3—Mg4^ix^	63.15 (12)	Mg2^iii^—Mg4—Zn3^viii^	59.58 (16)
Mg3—Zn3—Mg4^ix^	115.37 (12)	Zn3^vii^—Mg4—Zn3^viii^	52.13 (11)
Zn2—Zn3—Mg4^v^	62.78 (11)	Zn1^ii^—Mg4—Zn3^iii^	144.48 (15)
Zn3^vii^—Zn3—Mg4^v^	113.70 (12)	Zn1^iii^—Mg4—Zn3^iii^	93.19 (7)
Zn3^ix^—Zn3—Mg4^v^	64.55 (12)	Zn1—Mg4—Zn3^iii^	115.41 (10)
Zn2^ix^—Zn3—Mg4^v^	114.31 (12)	Al1—Mg4—Zn3^iii^	115.41 (10)
Zn3^x^—Zn3—Mg4^v^	117.80 (9)	Zn2^ii^—Mg4—Zn3^iii^	144.36 (19)
Mg1^vii^—Zn3—Mg4^v^	71.72 (13)	Zn2^iii^—Mg4—Zn3^iii^	50.59 (5)
Mg2^vii^—Zn3—Mg4^v^	121.16 (16)	Mg2^iii^—Mg4—Zn3^iii^	59.80 (9)
Mg2—Zn3—Mg4^v^	58.97 (10)	Zn3^vii^—Mg4—Zn3^iii^	50.71 (7)
Mg3^xi^—Zn3—Mg4^v^	115.64 (14)	Zn3^viii^—Mg4—Zn3^iii^	97.26 (15)
Mg3—Zn3—Mg4^v^	62.93 (13)	Zn1^ii^—Mg4—Zn3^ii^	93.19 (7)
Mg4^ix^—Zn3—Mg4^v^	178.26 (11)	Zn1^iii^—Mg4—Zn3^ii^	144.48 (15)
Al2—Al3—Mg2	109.42 (14)	Zn1—Mg4—Zn3^ii^	115.41 (10)
Mg2^vii^—Al3—Mg2	112.84 (5)	Al1—Mg4—Zn3^ii^	115.41 (10)
Mg2^vii^—Al3—Mg3^xi^	114.38 (14)	Zn2^ii^—Mg4—Zn3^ii^	50.59 (5)
Mg2—Al3—Mg3^xi^	123.21 (14)	Zn2^iii^—Mg4—Zn3^ii^	144.36 (19)
Al2—Al3—Mg3	65.98 (10)	Mg2^iii^—Mg4—Zn3^ii^	59.80 (9)
Mg2^vii^—Al3—Mg3	123.16 (7)	Zn3^vii^—Mg4—Zn3^ii^	97.26 (15)
Mg2—Al3—Mg3	113.61 (8)	Zn3^viii^—Mg4—Zn3^ii^	50.71 (7)
Mg3^xi^—Al3—Mg3	60.8 (2)	Zn3^iii^—Mg4—Zn3^ii^	119.59 (18)
Al2—Al3—Mg4^ix^	116.38 (12)	Zn1^ii^—Mg4—Zn2	94.76 (15)
Mg2^vii^—Al3—Mg4^ix^	59.16 (14)	Zn1^iii^—Mg4—Zn2	94.76 (15)
Mg2—Al3—Mg4^ix^	122.67 (9)	Zn1—Mg4—Zn2	47.95 (10)
Mg3^xi^—Al3—Mg4^ix^	63.15 (12)	Zn2^ii^—Mg4—Zn2	118.41 (10)
Mg3—Al3—Mg4^ix^	115.37 (12)	Zn2^iii^—Mg4—Zn2	118.41 (10)
Al2—Al3—Mg4^v^	62.78 (11)	Mg2^iii^—Mg4—Zn2	100.4 (2)
Mg2^vii^—Al3—Mg4^v^	121.16 (16)	Zn3^vii^—Mg4—Zn2	50.33 (10)
Mg2—Al3—Mg4^v^	58.97 (10)	Zn3^viii^—Mg4—Zn2	50.33 (10)
Mg3^xi^—Al3—Mg4^v^	115.64 (14)	Zn3^iii^—Mg4—Zn2	95.31 (12)
Mg3—Al3—Mg4^v^	62.93 (13)	Zn3^ii^—Mg4—Zn2	95.31 (12)
Mg4^ix^—Al3—Mg4^v^	178.26 (11)		

Symmetry codes: (i) *x*, *y*, −*z*; (ii) −*z*, *x*, *y*; (iii) *z*, *x*, *y*; (iv) −*y*, *z*, −*x*; (v) *y*, *z*, *x*; (vi) −*x*, *y*, *z*; (vii) −*y*+1/2, −*z*+1/2, −*x*+1/2; (viii) *y*−1/2, −*z*+1/2, −*x*+1/2; (ix) −*z*+1/2, −*x*+1/2, −*y*+1/2; (x) *x*, −*y*+1, *z*; (xi) −*x*+1/2, −*y*+1/2, −*z*+1/2; (xii) −*x*+1, −*y*+1, −*z*; (xiii) *z*+1/2, *x*+1/2, *y*−1/2; (xiv) *x*+1/2, *y*+1/2, −*z*+1/2; (xv) −*x*+1/2, −*y*+1/2, *z*−1/2; (xvi) *y*, −*z*+1, *x*; (xvii) −*z*+1/2, −*x*+1/2, *y*−1/2; (xviii) −*z*+1/2, *x*+1/2, −*y*+1/2; (xix) −*z*+1/2, *x*+1/2, *y*−1/2; (xx) *x*, −*y*+1, −*z*.

## References

[bb2] Berthold, R., Kreiner, G., Burkhardt, U., Hoffmann, S., Auffermann, G., Prots, Y., Dashjav, E., Amarsanaa, A. & Mihalkovic, M. (2013). *Intermetallics*, **32**, 259–273.

[bb3] Brandenburg, K. & Putz, H. (2017). *DIAMOND*. Crystal Impact GbR, Bonn, Germany.

[bb4] Bruker (2015). *APEX3* and *SAINT*. Bruker AXS Inc. Madison, Wisconsin, USA, 2008.

[bb5] Edoziuno, F. O., Adediran, A. A., Emereje, P. O., Akaluzia, R. O. & Jen, T. C. (2024). *Results Eng.* **21**, 101632.

[bb6] Krause, L., Herbst-Irmer, R., Sheldrick, G. M. & Stalke, D. (2015). *J. Appl. Cryst.* **48**, 3–10. 10.1107/S1600576714022985 PMC4453166 26089746

[bb7] Liu, C. & Fan, C. (2018). *IUCrData*, **3**, x180363.

[bb8] Montagné, P. & Tillard, M. (2016). *J. Alloys Compd.* **656**, 159–165.

[bb9] Sheldrick, G. M. (2015*a*). *Acta Cryst.* A**71**, 3–8.

[bb10] Sheldrick, G. M. (2015*b*). *Acta Cryst.* C**71**, 3–8.

[bb11] Westrip, S. P. (2010). *J. Appl. Cryst.* **43**, 920–925.

[bb12] Yao, C., Lv, H., Zhu, T., Zheng, W., Yuan, X. & Gao, W. (2016). *J. Alloys Compd.* **670**, 239–248.

[bb13] Zhang, Y., Wang, B., Wei, S., Wang, Y. & Li, L. (2022). *Heliyon*, **8**, e11224. 10.1016/j.heliyon.2022.e11224 PMC9618979 36325136

